# Hyperuricemia and overexcretion of uric acid increase the risk of simple renal cysts in type 2 diabetes

**DOI:** 10.1038/s41598-017-04036-6

**Published:** 2017-06-19

**Authors:** Ying Han, Mingliang Zhang, Junxi Lu, Lei Zhang, Junfeng Han, Fangya Zhao, Haibing Chen, Yuqian Bao, Weiping Jia

**Affiliations:** 1 Department of Endocrinology and Metabolism, Shanghai Jiaotong University Affiliated Sixth People’s Hospital, Shanghai Diabetes Institute, Shanghai Key Laboratory of Diabetes Mellitus, Shanghai Clinical Center for Diabetes, Shanghai, 200233 China; 2 Department of Endocrinology and Metabolism, Shanghai Eighth People’s Hospital, Shanghai, 200235 China

## Abstract

Previous studies have discussed the relationship between simple renal cysts (SRC) and serum uric acid level in healthy individuals. We performed a cross-sectional study to evaluate the association between serum uric acid level and fractional excretion of uric acid (FEUA) and simple renal cysts in males and postmenopausal females with type 2 diabetes. The overall prevalence of SRC was 18.1% in our population. SRC prevalence was significantly higher in hyperuricemic than normouricemic subjects (27.3% vs. 16.8%, P < 0.001). Subjects who overexcreted uric acid had a higher prevalence of SRC than underexcretors (total population: 21.6% vs. 16.3%; normouricemic subjects: 19.8% vs. 13.7%; hyperuricemic subjects: 50.0% vs. 22.7%, all P-values < 0.05). Hyperuricemia (odds ratio [OR] 1.824, 95% confidence interval [CI] 1.332–2.498, P < 0.001); FEUA (OR 1.046, 95% CI 1.002–1.091, P < 0.05); male gender (OR 1.922, 95% CI 1.489–2.480, P < 0.001); age (OR 1.049, 95% CI 1.035–1.064, P < 0.001); and albuminuria (OR 1.492, 95% CI 1.176–1.892, P < 0.01) were independent risk factors for SRC development. These findings suggested that hyperuricemia and high level of FEUA were both independent risk factors for SRC development in males and postmenopausal females with type 2 diabetes. Half of overproduction hyperuricemic patients had SRC.

## Introduction

Simple renal cysts (SRC) are the most common non-hereditary type of benign renal cysts in adults. The prevalence of SRC varies by population, geographic region, and the imaging modality used. Most SRC are asymptomatic, being accidentally detected by abdominal ultrasonography or computed tomography during a medical check-up or when another medical condition is being evaluated^1^. However, some cysts tend to increase in size and can be sufficiently large to cause pain, hematuria, and/or urinary obstruction^2, 3^. Also, previous studies showed that SRC may be associated with deterioration of renal function^4–6^. SRC presence correlated significantly with higher serum creatinine concentrations and reduced estimated creatinine clearances^5^. The prevalence of chronic renal failure in patients with SRC was about 1.5-fold greater than that in those lacking SRC^6^. SRC have also been suggested to be associated with hypertension^7, 8^; blood pressure normalization was reported after adequate treatment of SRC^9^. In general populations, all of poor renal function, hypertension, age^1, 10^, male gender^1, 10–13^, obesity^14, 15^, smoking^13^, renal stones^12, 13^ and serum uric acid level^16, 17^ are associated with SRC. It is important to identify (and eliminate) risk factors for SRC development. This would reduce the prevalence of the condition and improve patient outcomes.

Uric acid is a product of the metabolic breakdown of purine nucleotides and is excreted largely via the kidneys. Any relationship between serum uric acid level and SRC remains controversial. The prevalence of SRC was significantly higher in a gout group than in a sex- and age-matched control group (26.0% vs. 10.6%)^16^. Serum uric acid is the most important factor in gout pathogenesis, and a high serum uric acid level was significantly associated with the presence of SRC^17^. However, some authors consider that no significant independent association is evident between serum uric acid level and SRC^4, 15, 18^. No study has addressed the relationship between fractional excretion of uric acid (FEUA) and SRC prevalence.

The number of patients with type 2 diabetes is increasing markedly. Some authors found that diabetes was not significantly associated with SRC development^8, 14, 18^. Others reported significant increases in the prevalence of diabetes (9.7–24.1%) in patients with SRC, but this was not significant upon multivariate modeling^1, 12, 15, 19^. Therefore, diabetes per se was not a significant independent risk factor for the development of SRC, but other factors associated with diabetes may be relevant. For example, uric acid, the serum level of which may be associated with SRC development, is processed differently by the kidneys of diabetic patients and healthy individuals^20^. A previous study suggested that type 2 diabetic patients may experience either hypouricemia or hyperuricemia^21^. To our knowledge, no association between uric acid level and SRC in type 2 diabetic patients has yet been reported. It is important to study the possible relationships between serum uric acid level and FEUA and SRC in type 2 diabetic patients, in an effort to reduce SRC development.

## Results

### Characteristics of subjects with and without SRC

The clinical characteristics of males and postmenopausal females with type 2 diabetes, with and without SRC, are shown in Table 1. Diabetics with SRC were more likely to be elderly males. Diabetics with SRC exhibited a longer duration of diabetes; higher serum uric acid and serum creatinine level; and a greater FEUA; and lower eGFR, alanine aminotransferase, aspartate aminotransferase, γ-glutamyl transferase, total cholesterol, high-density lipoprotein cholesterol, low-density lipoprotein cholesterol, and fasting plasma glucose level, compared with diabetics without SRC. Accompanied diseases and background therapies of subjects with and without SRC are shown in Table 2. Diabetic nephropathy, hypertension and urolithiasis were more common in subjects with SRC.

**Table 1:** Clinical characteristics of subjects with and without SRC.

	With SRC(n = 839)	Without SRC(n = 3786)	P-value
Age (years)	64.43 ± 10.40	59.65 ± 11.74	<0.001
Men (n, %)	579, 69.0%	2208, 58.3%	<0.001
BMI (kg/m^2^)	25.22 ± 3.55	25.02 ± 3.59	0.150
DD (years)	11.15 ± 7.22	10.35 ± 7.27	0.005
Albuminuria (n, %)	290, 35.8%	958, 26.2%	<0.001
SUA (µmol/L)	336.80 ± 97.37	320.79 ± 85.93	<0.001
SCr (µmol/L)	81.46 ± 52.54	70.37 ± 25.86	<0.001
eGFR (mL/min/1.73 m^2^)	93.60 ± 30.01	102.82 ± 28.26	<0.001
ALT (U/L)	17 (12–26)	19 (14–29)	<0.001
AST (U/L)	18 (15–23)	19 (15–24)	0.021
γ-GT (U/L)	24 (17–36)	25 (18–40)	0.016
TC (mmol/L)	4.56 ± 1.14	4.74 ± 1.17	<0.001
TG (mmol/L)	1.34 (0.91–1.89)	1.35 (0.94–2.01)	0.247
HDL-C (mmol/L)	1.06 ± 0.29	1.09 ± 0.30	0.017
LDL-C (mmol/L)	2.69 ± 0.91	2.83 ± 0.88	<0.001
FPG (mmol/L)	7.66 ± 2.51	7.93 ± 2.91	0.007
HbA1C (%)	8.68 ± 2.22	8.64 ± 2.05	0.604
24-h UUA (µmol)	3034.36 ± 1515.62	2980.05 ± 1314.67	0.396
24-h UCr (µmol)	10607.07 ± 5604.81	10377.53 ± 5378.55	0.373
24-h UGLU (mmol)	6.32 (0.59–40.18)	7.81 (0.51–42.82)	0.691
FEUA (%)	6.92 ± 2.73	6.64 ± 2.50	0.033

Continuous variables of normal distribution were expressed as mean ± SD and compared by the Student’s t test. Non-normal distribution variables were expressed as and median (interquartile range 25–75%) and compared by the Mann-Whitney U test. Categorical variables were expressed as percentages and compared by the Chi-square test.

SRC, simple renal cysts; BMI, body mass index; DD, duration of diabetes; SUA, serum uric acid; SCr, serum creatinine; eGFR, estimated glomerular filtration rate; ALT, alanine aminotransferase; AST, aspartate aminotransferase; γ-GT, γ-glutamyl transferase; TC, total cholesterol; TG, total triglycerides; HDL-C, high-density lipoprotein cholesterol; LDL-C, low-density lipoprotein cholesterol; FPG, fasting plasma glucose; HbA1c, glycosylated hemoglobin; 24-h UUA, 24-hour urine uric acid; 24-h UCr, 24-hour urine creatinine; 24-h UGLU, 24-hour urine glucose; FEUA, fraction excretion of uric acid.

**Table 2:** Accompanied diseases and background therapies of subjects with and without SRC.

	With SRC(n = 839)	Without SRC(n = 3786)	P-value
DPN (n, %)	200, 23.8%	832, 22.0%	0.241
DR (n, %)	196, 23.4%	858, 22.7%	0.662
DN (n, %)	230, 27.4%	786, 20.8%	<0.001
PVD (n, %)	596, 71.0%	2630, 69.5%	0.370
Urolithiasis (n, %)	115, 13.7%	376, 9.9%	0.001
Hypertension (n, %)	542, 64.6%	2073, 54.8%	<0.001
Hyperlipidemia (n, %)	250, 29.8%	1233, 32.6%	0.120
Hypertriglyceridemia (n, %)	93, 11.1%	420, 11.1%	0.739
Hypercholesterolemia (n, %)	90, 10.7%	444, 11.7%	0.298
Combined hyperlipidemia (n, %)	67, 8.0%	369, 9.7%	0.088
CHD (n, %)	112, 13.3%	453, 12.0%	0.268
Stroke (n, %)	107, 12.8%	434, 11.5%	0.293
Anti-diabetic drug users (n, %)	772, 92.0%	3416, 90.2%	0.109
Monotherapy (n, %)	184, 21.9%	781, 20.6%	0.091
Bitherapy (n, %)	277, 33.0%	1221, 32.3%	0.129
Multitherapy (n, %)	311, 37.1%	1414, 37.3%	0.185
Anti-hypertensive drug users (n, %)	214, 39.5%	808, 39.0%	0.830
ACEI users (n, %)	26, 4.8%	107, 5.2%	0.775
ARB users (n, %)	188, 34.7%	701, 33.8%	0.743

Categorical variables were expressed as percentages and compared by the Chi-square test.

SRC, simple renal cysts; DPN, diabetic peripheral neuropathy; DR, diabetic retinopathy; DN, diabetic nephropathy; PVD, peripheral vascular disease; CHD, coronary heart disease; ACEI, angiotension converting enzyme inhibitors; ARB, angiotensin receptor blocker.

### Comparison of SRC status among groups with different serum uric acid levels

**Figure 1:**
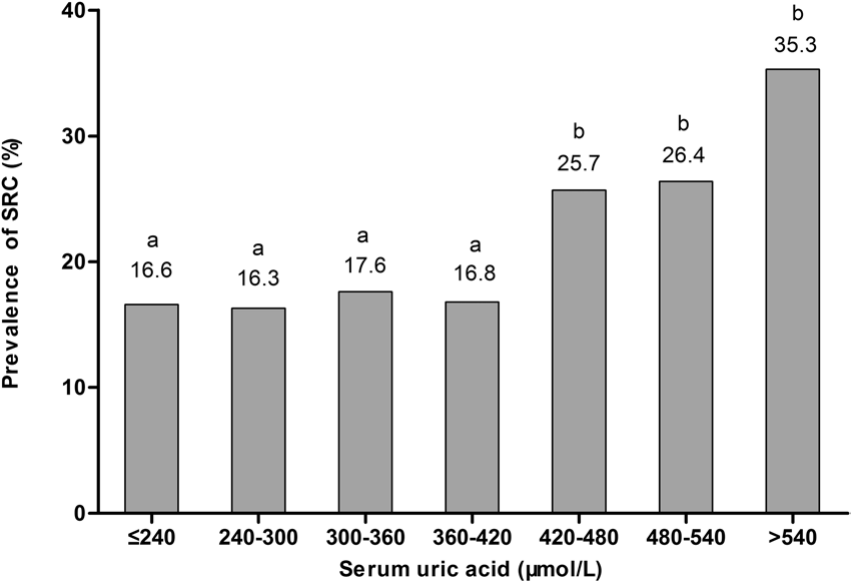
Simple renal cysts (SRC) prevalence in groups with different serum uric acid levels. The subjects were divided into seven groups by 60-µmol/L increments in serum uric acid (no further stratification for subjects with serum uric acid level ≤240 µmol/L or >540 µmol/L). The P-value was <0.05 when the a and b groups were compared.

The SRC status of groups with different serum uric acid levels is shown in Fig. 1. SRC prevalence increased when the serum uric acid was >420 µmol/L. Each successive group with a higher serum uric acid level (>420 μmol/L) had a significantly higher prevalence of SRC than did the group below (all P-values < 0.05).

### Comparison of SRC status among different FEUA groups

**Figure 2:**
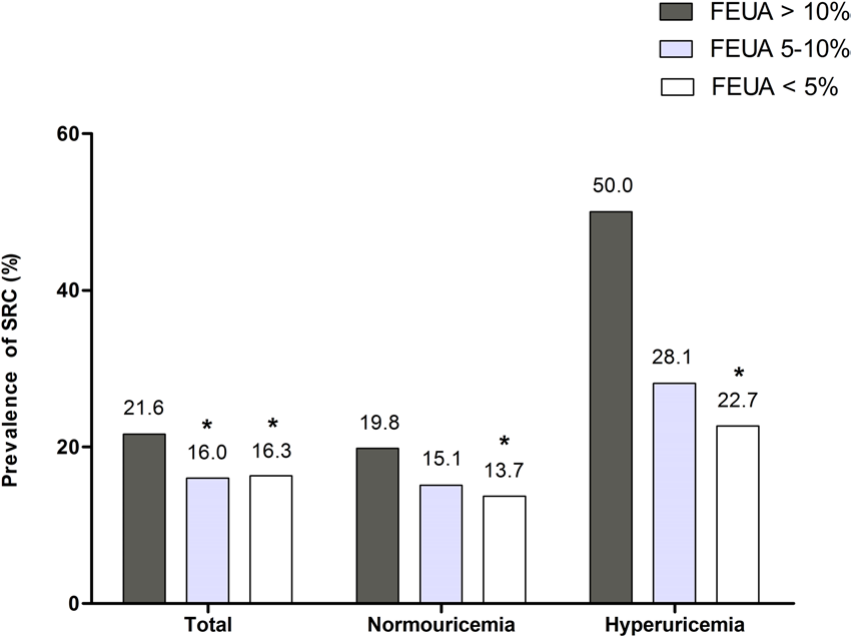
Simple renal cysts (SRC) prevalence among different fractional excretions of uric acid (FEUAs) groups in the total population, and in hyperuricemic and normouricemic subjects. The subjects were divided into three groups (FEUA >10%, 5–10%, and <5%). *P < 0.05 compared with the FEUA >10% group.

A comparison of SRC status among different FEUA groups is shown in Fig. 2. The subjects were stratified into three groups: overexcretion of uric acid (FEUA: >10%), normal-excretion of uric acid (FEUA: 5–10%), and underexcretion of uric acid (FEUA: <5%). In the total population, the prevalence of SRC in the overexcretion group (21.6%) was significantly higher than that in the other two groups (16.0% and 16.3%, both P-values < 0.05). Upon stratification analysis, the overexcretion group had a higher prevalence of SRC than the underexcretion group; this was true of both normouricemic and hyperuricemic subjects (normouricemic subjects: 19.8% vs. 13.7%; hyperuricemic subjects: 50.0% vs. 22.7%, both P-values < 0.05).

### Independent risk factors for SRC development

All variables with P-values < 0.05 in Table 1 and Table 2 (except serum uric acid, eGFR, and diabetic nephropathy), including hyperuricemia, age, sex, duration of diabetes, albuminuria, serum creatinine, alanine aminotransferase, aspartate aminotransferase, γ-glutamyl transferase, total cholesterol, high-density lipoprotein cholesterol, low-density lipoprotein cholesterol, fasting plasma glucose, FEUA, hypertension and urolithiasis were selected as covariates in multivariate logistic regression to identify risk factors for SRC development. In males and postmenopausal females with type 2 diabetes, hyperuricemia (odds ratio [OR] 1.824, 95% confidence interval [CI] 1.332–2.498, P < 0.001); FEUA (OR 1.046, 95% CI 1.002–1.091, P < 0.05); male gender (OR 1.922, 95% CI 1.489–2.480, P < 0.001); age (OR 1.049, 95% CI 1.035–1.064, P < 0.001); and albuminuria (OR 1.492, 95% CI 1.176–1.892, P < 0.01) were independent risk factors for SRC development (Table 3).

**Table 3:** Univariate and multivariate logistic regression of factors associated with SRC.

	Unadjusted odds ratio (95% confidence intervals)	P-value	Adjusted OR (95% CI)	P-value
Hyperuricemia	1.869 (1.529–2.285)	<0.001	1.824 (1.332–2.498)	<0.001
Age	1.039 (1.032–1.047)	<0.001	1.049 (1.035–1.064)	<0.001
Men	1.592 (1.356–1.868)	<0.001	1.922 (1.489–2.480)	<0.001
DD	1.015 (1.005–1.025)	<0.01	—	
Albuminuria	1.572 (1.337–1.847)	<0.001	1.492 (1.176–1.892)	<0.01
SCr	1.010 (1.007–1.013)	<0.001	—	
ALT	0.993 (0.989-.997)	<0.01	—	
AST	0.992 (0.986–0.999)	<0.05	—	
γ-GT	0.999 (0.998–1.001)	0.318	—	
TC	0.866 (0.808–0.928)	<0.001	—	
HDL-C	0.727 (0.560–0.944)	<0.05	—	
LDL-C	0.834 (0.764–0.910)	<0.001	—	
FPG	0.965 (0.938–0.993)	<0.05	—	
FEUA	1.041 (1.005–1.079)	<0.05	1.046 (1.002–1.091)	<0.05
Hypertension	1.508 (1.291–1.761)	<0.001	—	
Urolithiasis	1.441 (1.152–1.802)	<0.01	—	

SRC, simple renal cysts; DD, duration of diabetes; SCr, serum creatinine; ALT, alanine aminotransferase; AST, aspartate aminotransferase; γ-GT, γ-glutamyl transferase; TC, total cholesterol; HDL-C, high-density lipoprotein cholesterol; LDL-C, low-density lipoprotein cholesterol; FPG, fasting plasma glucose; FEUA, fraction excretion of uric acid.

## Discussion

We found that SRC prevalence increased sharply when the serum uric acid level was >420 µmol/L in males and postmenopausal females with type 2 diabetes. This serum uric acid level was the lower limit for diagnosis of hyperuricemia. The prevalence of SRC was 27.3% in hyperuricemic and 16.8% in normouricemic subjects. Subjects who overexcreted uric acid had a higher prevalence of SRC than underexcretors (in the total population: 21.6% vs. 16.3%; in normouricemic subjects: 19.8% vs. 13.7%; in hyperuricemic subjects: 50.0% vs. 22.7%). We found that hyperuricemia independently increased the risk of SRC by 82.4%. For every percentage rise in FEUA, the risk of SRC increased approximately by 4.6%. Male gender (OR 1.922, 95% CI 1.489–2.480), age (OR 1.049, 95% CI 1.035–1.064) and albuminuria (OR 1.492, 95% CI 1.176–1.892) were also independent risk factors for SRC development.

The overall prevalence of SRC is 4–12% in general populations^1, 10, 12, 13, 18, 19^. In the present study, the figure was 18.1%, thus higher than those of other studies. In the cited works, the mean age ranged from 40–70 years and the proportion of males from 55–70%, similar to our figures. SRC were detected by ultrasonography in both earlier studies and our current study. But we evaluated males and postmenopausal females with type 2 diabetes; earlier works studied healthy individuals. This may explain why our SRC prevalence was high. Factors associated with the diabetic state may increase the prevalence of SRC. However, the etiology of SRC formation remains unclear in both diabetics and others. Some authors have suggested that SRC are derived from diverticulae of the collecting or distal convoluted tubule. Such diverticulae may develop upon weakening of the tubular basement membrane^22, 23^.

Any association between serum uric acid level and SRC development was previously controversial. Two case-control studies found no significant differences between SRC patients and controls^4, 18^. Cross-sectional studies on healthy subjects and internal medicine outpatients indicated that serum uric acid level were associated with SRC development upon univariate analysis^14, 15^, and, indeed, even upon multivariate analysis adjusted for confounders including age, sex, eGFR, and renal stones^17^. Blijderveen *et al*. suggested that, in females, the risk of SRC increased by 78% as the serum uric acid level rose (from about 113 to 660 µmol/L), and that males with serum uric acid level <240 µmol/L were at a significantly lower risk of SRC development compared with those with level ≥240 µmol/L^17^. Therefore, we suggested that uric acid may damage the renal tubules, which may in turn trigger SRC formation. One animal experiment has shown that uric acid injures the tubules^24^.

However, we found that SRC prevalence did not rise until the serum uric acid level was >420 µmol/L. This is the saturation level; above this level, monosodium urate crystals may form. Thus, chronic hyperuricemia may cause urate crystals to be deposited in many tissues, including intrarenal tissues. Animal model studies have shown that monosodium urate precipitates in the tubular lumen can erode the tubular basement membrane, pass into the interstitium, and promote inflammation and fibrosis therein^25^. Therefore, it is possible that monosodium urate crystals, rather than soluble uric acid, weaken the tubular basement membrane and trigger SRC formation.

To our knowledge, no relationship between uric acid excretion and SRC status has yet been reported in either a general or diabetic population. In our current study, subjects who overexcreted uric acid had a higher prevalence of SRC than those underexcreted uric acid, high FEUA was an independent risk factor for SRC development. A higher FEUA means a larger proportion of uric acid excreted into urine and a greater possibility of urate crystals being deposited in urinary system. And excessive uric acid excretion is often due to the excessive production of uric acid, the prevalence of SRC in hyperuricemic overexcretors (those with “overproduction hyperuricemia”) was as high as 50%. Thus, patients with overproduction hyperuricemia are at particular risk of SRC development.

The different means by which type 2 diabetic patients and healthy individuals process renal uric acid may explain the difference in SRC prevalence between our study and other studies. Renal uric acid processing has four steps: glomerular filtration of virtually all circulating uric acid, pre-secretory reabsorption, secretion, and post-secretory reabsorption^26^. The situation may be much more complex in diabetic patients. On one hand, osmotic diuresis caused by high plasma glucose level may enhance uric acid excretion^27^. In addition, in early diabetic kidney disease, the GFR may increase before the appearance of nephropathic clinical symptoms and signs. Glomerular hyperfiltration may increase uric acid clearance. On the other hand, the increased proximal reabsorption of glucose may exacerbate uric acid retention^28^. In addition, hyperinsulinemia developing secondary to insulin-resistance may impair the renal clearance of uric acid^29^. It is also possible that complexities associated with urate excretion in diabetics explain the results in our study.

Although most SRC are benign and usually not an indication for active therapeutic measures, interventions are needed when accompanied by conditions such as hydronephrosis with pressure atrophy of the renal parenchyma, pain caused by the cyst, and deteriorating renal function^30^. In addition, blood pressure normalization was reported after adequate treatment of SRC^9^. We should give enough attention to SRC. Hyperuricemia and high level of FEUA were both closely related to SRC. Therefore, even asymptomatic hyperuricemia requires standardized uric acid lowering treatment to prevent the occurrence of SRC. In addition, urolithiasis, but not SRC, was one of the contraindications of uricosuric drugs in the past. However, according to our results, SRC patients should also be careful to use uricosuric drugs.

Our study had certain limitations. We evaluated males and postmenopausal females with type 2 diabetes; our results may thus not be generalizable to other populations. In addition, the cross-sectional nature of the study allows us to infer (only) temporal relationships between uric acid level and SRC status. Further prospective studies are required to explore causal relationships.

In conclusion, our results suggested that in Chinese males and postmenopausal females with type 2 diabetes, hyperuricemia, high level of FEUA, male gender, age and albuminuria were independent risk factors for SRC development. Half of overproduction hyperuricemic patients had SRC. Therefore, type 2 diabetes patients with hyperuricemia, and/or a high FEUA, are at risk of SRC. No study has yet assessed the prevalence of SRC in hyperuricemic or gout patients taking uricosuric agents. A prospective study is needed to explore whether uricosuric agents would affect the incidence of SRC.

## Methods

### Subjects

**Figure 3:**
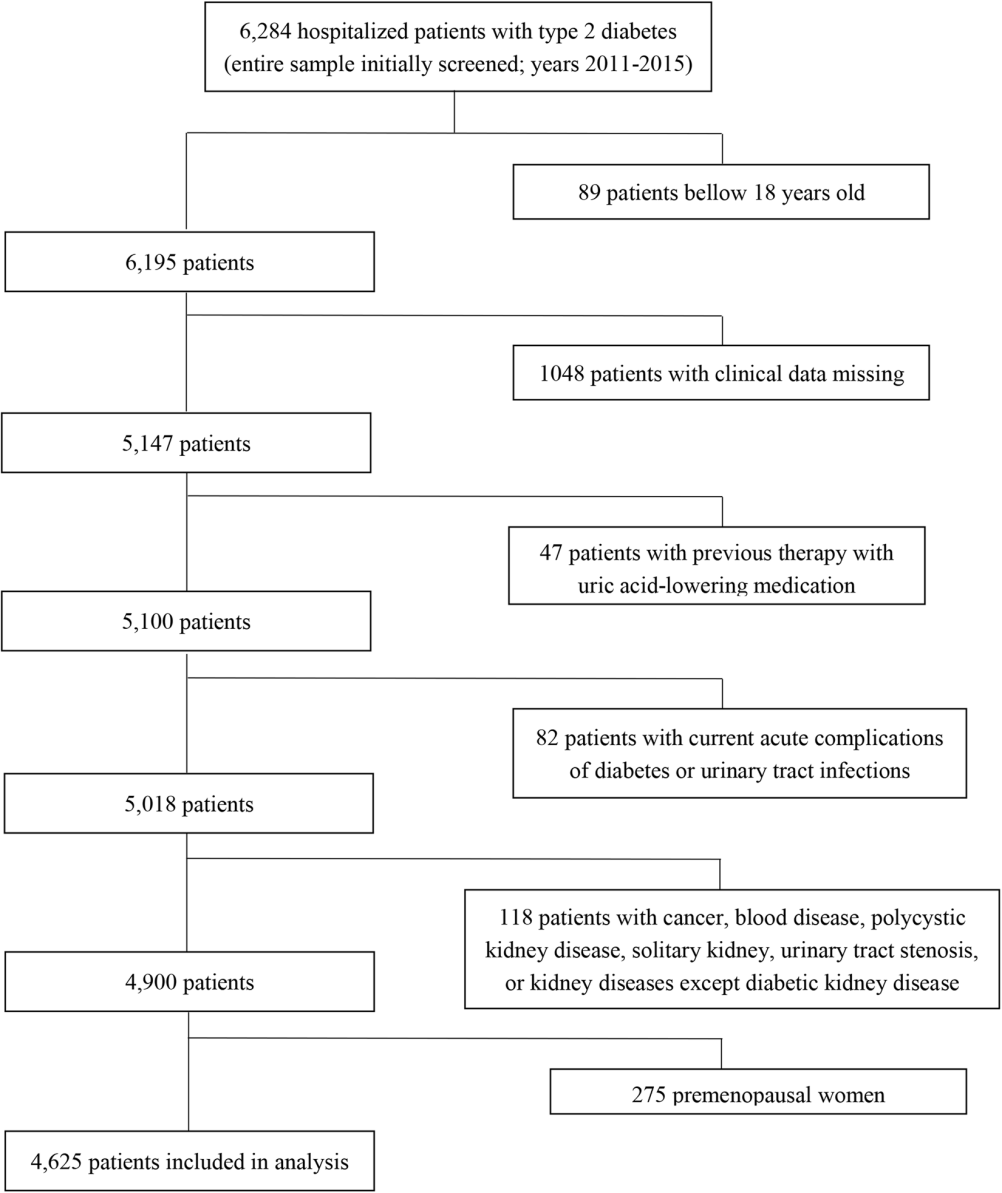
Details of the study design.

A Total of 6,284 hospitalized Chinese type 2 diabetes patients was observed between 2011 and 2015. All subjects were diagnosed with type 2 diabetes mellitus based on the 1999 criteria of the World Health Organization. Other types of diabetes and following patients were excluded from the study: (i) bellow 18 years old; (ii) clinical data was incomplete; (iii) previous therapy with uric acid-lowering medication; (iv) patients with current acute complications of diabetes or urinary tract infections; (v) patients with cancer, blood disease, polycystic kidney disease, solitary kidney, urinary tract stenosis, or kidney diseases except diabetic kidney disease. A total of 1,384 subjects were excluded. We found that the most of hospitalized patients with type 2 diabetes were elderly patients, and the gender differences of uric acid metabolism was mainly between men and premenopausal women. So we excluded 275 premenopausal women for ease of analysis. Finally, 4,625 subjects were included in this cross-sectional study (Fig. 3). The study was performed according to the principles of the Declaration of Helsinki and was approved by the ethics committee of Shanghai Jiao Tong University Affiliated Sixth People’s Hospital (#2011–32). All study subjects provided informed consent.

### Clinical measurements

Age, sex, accompanied diseases, duration of diabetes and background therapies for diabetes (monotherapy, bitherapy and multitherapy) and hypertension (only including ACEI and ARB) were recorded in everyone. The height and weight of the subjects were measured. Their body mass index (BMI) was calculated as weight (kg)/height (m)^2^. All subjects were on a normal purine diet for at least 3 days before collecting blood and urine samples. Venous blood specimens were obtained after an overnight fasting for measuring the serum concentrations of uric acid, creatinine, alanine aminotransferase, aspartate aminotransferase, γ-glutamyl transferase, total cholesterol, total triglycerides, high-density lipoprotein cholesterol, low-density lipoprotein cholesterol, glucose, and glycosylated hemoglobin. A single 24-hour urine sample was collected for measuring the 24-hour urine uric acid, creatinine, glucose and microalbuminuria. Serum concentrations of uric acid, creatinine, alanine aminotransferase, aspartate aminotransferase, γ-glutamyl transferase, and lipids, including total cholesterol, triglycerides, high-density lipoprotein cholesterol and lowdensity lipoprotein cholesterol were measured using a type 7600–020 automated analyzer (Hitachi, Tokyo, Japan). Glucose concentrations were measured by the glucose oxidaseperoxidase method using commercial kits (Shanghai Biological Products Institution, Shanghai, China). Glycosylated hemoglobin was determined using the Bio-Rad VARIANT II analyzer (Bio-Rad Laboratories, Hercules, CA). Urinary concentrations of uric acid and creatinine were measured on a Hitachi 7600 analyzer using the sarcosine oxidase PAP method. The estimated glomerular filtration rate (eGFR) was calculated from the four-variable modification of diet in renal disease (MDRD) equation: eGFR (ml/min/1.73 m^2^) = 186.3 × [serum creatinine (µmol/L)/88.4]^−1.154^ × [age(year)]^−0.203^ × (0.742 if female) × (1.21 if black)^31^. FEUA (%) = [serum creatinine (µmol/L) × 24-hour urine uric acid (µmol)]/[(serum uric acid (µmol/L) × 24-hour urine creatinine (µmol)] × 100%. Albuminuria was defined as 24h-hour urine microalbuminuria of 30 mg and higher.

Hyperuricemia was diagnosed when serum uric acid level was >420 µmol/L. Ultrasonography examinations were performed using an IU 22 scanner equipped with a linear 3–7 MHz probe. SRC were diagnosed by experienced radiologists. The sonographic criteria for the diagnosis of simple renal cysts included absent internal echoes, a smooth, sharply defined wall, and posterior acoustic enhancement, indicating posterior through transmission that was not suspicious for a malignant renal mass.

### Statistical Analyses

SPSS 20.0 for windows was used for analyzing the study data. Normality was checked using the Kolmogorov-Smirnov test for continuous variables. The continuous variables of normal distribution were expressed as mean ± SD and compared by the Student’s t test. The non-normal distribution variables were expressed as and median (interquartile range 25–75%) and compared by the Mann-Whitney U test. The categorical variables were expressed as percentages and compared by the Chi-square test. The binary logistic regression analysis was used to determine the independent risk factors of SRC. The variables selected to enter into binary regression were those that correlated significantly with SRC (P < 0.05) by Student’s t test, Mann-Whitney U test and Chi-square test. For all analyses, P-value less than 0.05 were considered to be statistically significant.
